# Uterine Surgery

**Published:** 1868-03

**Authors:** C. C. F. Gay

**Affiliations:** Buffalo


ART. III.—*Uterine Surgery.* By C. C. F. Gay, M. D., *Buffalo.*

To learn anything new and to become acquainted with any new theory or principle involves an exercise of the mind somewhat difficult, and a task sometimes hard of attainment, but it is harder and still more difficult to forget past errors and to unlearn that which has been already learned or accepted and practiced for a series of years, as principles established and long held to be trustworthy, without a passing thought that they might be false. Have we anything to unlearn or forget in the treatment of uterine diseases? Can it be shown that our theory has been founded upon a false pathology, and that such theory has led to erroneous treatment? I think a proposition of this character susceptible of demonstration, and therefore propose, in writing this paper, that one of its objects shall be to discuss this proposition, and attempt to show as clearly as I am able that the practitioner of the present day who has either occasionally practiced his art in this department of medicine, or has indeed made it in some degree a specialty, must now lay old theories aside, dispose of his preconceived ideas as best he may, put away his old instruments to rust, and, in a word, begin *de novo* to study up the true pathology and treatment of uterine disease. It will be conceded a difficult task to lay by an old instrument with the use of which we have become habituated from long employment, and take up another with which much time is required to familiarize ourselves to its dextrous management, but it has come to this, and the fact had better at once be conceded, and promptly acted upon, if we desire to keep ourselves *au courant* with the progress of our times, and dispense the largest amount of good to our disabled race in the shortest possible space of time. This branch of special practice must be learned over again, we must start out on our journey anew, if we do not tacitly consent to be left to loiter by the way-side, or to be left with the straglers in the rear of the army of men of progressive ideas.

Dislocation of the fundus uteri, anteriorly or posteriorly, constituting that departure from its normal condition, called antiversion and retroversion, is not a cause *per se* of much pain or disability to the female, only as the fundus impinges upon the bladder or rectum, or becomes either engorged or congested, and occludes the cervix, producing dismenorrhœa. Diagnosis of these displacements has always been easy and made latterly by the introduction of Simpson's sound, an instrument which causes much pain, and is not always unattended with danger of producing pelvic cellulitis. This instrument must be abandoned and the silver probe substituted in its place—not however for the purpose of being used as a lever upon which to replace the fundus in its normal position, for experience has shown, I think, that the fundus will resume its former position as often as the sound has placed the fundus in its normal position, but the silver probe should be used, simply to ascertain the direction which the curvature of the cervix takes.

Antiflexion may be diagnosed without the use of the sound most easily and correctly; the female with an antiflexed uterus will have a pain at the top of her head, which may be regarded as pathognomonic of antiflexion; she will *look* and *act* antiflected, so that one may diagnose this displacement correctly by the physico-physiognomy of the patient. The relative number of antiflexions and retroversions, I suppose is unknown, probably one of the former to fifteen or twenty of the latter. These displacements constitute at present the approbria of the specialist in this department of medicine; nothing but failure has as yet attended the efforts of the most skillful to restore permanently the uterus to its normal position, and nothing but failure will attend all our best directed efforts, until other means than those at present devised, are brought into requisition. The intra-uterine pessary has been tried and found wanting; the cotton pessary, made of immense size, and crowded up by the side of the cervix, and indeed the vagina tamponed with cotton, is no better; all these appliances and temporising plans of treatment must give way to something more effective. It is reserved for some surgeon to gain immortal honor and fame by the discovery of an operation which shall take the place of these temporising and unsatisfactory modes of treatment, and which shall be adequate to the radical cure of these displacements of the uterus.

That such a consummation, so devoutly to be wished, is within the range of probabilities, I need only cite the advance made by Sims in the surgical treatment of procidentia. While the efforts of our profession were directed in the way of inventing a pessary which should hold the hypertrophied cervix in place, and while these pessaries of divers forms and patterns have accumulated until if collected into one store-house, they would constitute a curious and cumbrous cabinet, the mind and efforts of Sims took a wider range; he originated an operation which Emmet has perfected, by which the os tincæ may be kept up in place without the aid of a pessary, the principal feature of the operation consisting in the diminution of the vaginal calibre.

It is not within the scope of this paper to more than thus cursorily pass in review the displacements of the uterus. The writer purposes in a future paper to resume the subject. Enough, however, has been said to accomplish the aims of the writer as set forth in the beginning of the paper, wherein he proposed to show that many of the appliances now in use for the supposed good of the female whose fundus uteri chooses to deviate from its normal and healthful position have given way to the more effectual procedure of operative interference.

The writer proposes to limit the residue of this paper to the subject of abrasions, congestions and ulcerations of the os uteri. This being the most common condition of the organ with which the physician has to deal, the treatment of these conditions most probably being the limit of the experience and practice of most physicians. The causes of these ulcerations will be left out of the paper entirely as not being pertinent and relevant to the discussion. We propose to examine the question under consideration practically, only, without proposing to enter into the merits or demerits of the subject from an etiological or pathological stand-point.

We assume that an abrasion or ulceration upon the os uteri, as presented to view through a cylindrical speculum, is amenable to cure in a very short time by appropriate local applications, and we likewise assume the position that when these ulcers, so-called, appear to be healed and cured after a two or three months' treatment, are in fact not cured at all in many instances, because the real seat of disease is higher up, has not been reached by our local applications, and hence the frequent relapses so soon after the patient has been discharged. We assume that the diseased portion of the uterus brought to view by this form of speculum is but a manifestation of that disease existing within or beyond the external or internal os, and that remedies to be effective must be carried within and through the cervix uteri.

Applications to the os of nitrate of silver have been made every four or five days, extending over a period of three months before we have been able to assure our patients that they need no further treatment. This method of treatment is comparatively modern, and has opened up the way for much imposition practiced on the part of the physician towards his patient. An ulcerated point may be produced upon the healthy os by a single application of the solid stick of argenti nitras, and after being thus produced may be healed in three days by a single application of glycerine, or by the use of the cotton pessary partially saturated with equal parts of iodine and ol. morrhuæ.

I have said that much imposition has been practiced upon patients by this method of local medication, not however by members of the regular profession, but by those impostors who infest our ranks, and in whom numerous persons, even of intelligence, are willing to intrust their lives and health. Such practitioners subject their patients to a long course of topical treatment, either through ignorance or love of money. The guilt of the transaction is alike in both cases, of equal enormity, and the names of such practitioners should be written down in the roll of those who love money more than they love their fellow-men, and they should be branded as impostors and quacks, and be known and acknowledged as such by all honorable men.

Leucorrhœa, a disease, or symptom of disease, so intractable to internal medication, has long been regarded as the result of diseased os, or cervix, and the speculum employed, and very properly employed at once, before wasting time in the use of a course of medication, in order to ascertain whether or not the discharge results from ulcerations of the os. Instruments heretofore employed have enabled us to reach only that diseased portion brought into view through the speculum. Sometimes, however, the solid stick of nitrate of silver has been made to penetrate a short distance within the cervix, but when, after a course of treatment, extending over a considerable period of time, the parts made visible have assumed healthy aspect, and the local disease appeared to be cured, it has been found that the leucorrhœal discharge continues as before. The explanation of this, in my opinion, is to be found in the fact that the seat of disease had not been reached by the applications made, owing to faulty construction of instruments, or rather to want of instruments properly constructed. To supply this deficiency the writer devised and had constructed some years since an instrument which measurably supplied the want. One-half the length of the instrument was made of copper, the other half of silver; the latter made to screw into the former; it was flexible, the end provided with a shallow groove, into which was poured smelted nitrate of silver. Indeed, construction of the instrument at its extremity was the same as that of the porte caustique; its size was one-eighth of an inch in diameter. This instrument, armed with the caustic, was bent at the required curve, and introduced directly into and through the cervix, answering a good purpose whenever the caustic was required to be employed, but for carrying into the uterine cavity any other medicinal agent it will be seen that the instrument was entirely useless. The instrument I now use is made of virgin silver, flattened and quite flexible, around the extremity of this instrument for a space of two inches from and including the point, a coating of cotton is entwined and the instrument dipped into any fluid preparation which the disease in hand requires, and then carried through the cervix to the fundus. This instrument or applicator, before using, is bent so as to adapt itself to the curve of the cervix, and in order to ascertain the required curve the silver flexible probe is first introduced and withdrawn, which gives the proper degree of curvature required of the applicator.

I believe the ordinary cylindrical speculum and the bivalve speculum have had their day, and that they must now be numbered among the things that were. Having been useful in their day they are of no longer use when a better instrument can supply their place. Sims' speculum, with the patient in the semi-prone position, is by all odds the best instrument yet invented for exclusive and general use, for all cases, whether involving operation or simply for topical applications, where the employment of the speculum is indicated. It unhappily possesses one demerit, in that it requires the aid of an assistant as a general rule. To surmount this defect Dr. Emmet of New York, has devised an instrument which is self-supporting; it is in all its essential features a Sims' speculum with Emmet's addition, and the addition is certainly no improvement, provided an assistant could always be at hand to properly hold the instrument of Sims. I am now using the Emmet speculum, and in the absence of an assistant it answers the purpose well. I much prefer it to the cylinder, but do not prefer it to the Sims' speculum when placed in the hands of a competent assistant. With this instrument *in situ* the mucous membrane of the os may be siezed with the tenaculum without causing any pain or hemorrhage, unless there be considerable engorgement, and drawn down to view and thus held until the applicator prepared as above described has been introduced and withdrawn from the cavity of the uterus.

After the topical application has been made it has been for a long time a custom of mine to introduce a ball of cotton wadding, so constructed as to possess a stem or pedicle, made by entwining thread around the stem. This stem serves as a convenient handle in removing the ball or cotton pessary. The cotton is moistened and sometimes saturated with glycerine and retained *in situ* from four to twenty-four hours, the time being regulated by the degree of annoyance it may cause the patient. All will concede, I think, that the treatment of these diseases of the os uteri has heretofore been somewhat irrational, if not empirical. The great want of the profession has been an instrument, by the use of which remedial agents might be carried directly in contact with diseased tissue. In the construction and use of the applicator, which is herein described, that want is supplied, and treatment made effectual. We need not now tell our patients that two or three months are required, when as many weeks are sufficient to restore the os uteri to a healthful condition.

The diseases appearing to our view upon the os uteri being only an index and sequence of disease within the cervix, treatment becomes rational when the abraided or ulcerated os is passed by, and remedies brought in direct contact with diseased tissue beyond. The tongue is protruded to give us an indication of the state of the mucous membrane of the stomach; if we find upon the tongue an aphthous condition or epithelial tissue destroyed, we do not expect to cure the disease by application of caustics or astringents to the tongue or the walls which encircle it, but knowing the condition of the tongue to be but an index of that pathological condition to be found in the mucous membrane of the stomach, if it could be seen, our remedies are addressed to the relief of this organ, and so soon as diseased tissue is made convertible into healthy tissue, the tongue resumes its normal aspect. Rational treatment here is in all respects what we should call rational treatment in cases under consideration.

I now desire to devote a little space in calling attention to the class of remedies usually accepted by the profession as appropriate for local application, and in regard to some remedies of this class, to speak somewhat in detail. A great variety of agents have been in demand, ranging from the light antiphlogistic touch with the crayon of caustic, to the powerful escharotics and actual cautery. Standing at the head of this class of remedial agents should be placed the chromic acid, whether there be much or little disease, this acid possesses the merit of attacking diseased tissue and non-interfering with healthy tissue, its caustic or escharotic properties being destroyed or suspended almost simultaneously with its application. The strength of the acid which I have found to be most desirable for use, is that made with three parts of water with one part by weight of the acid. When the lips of the os have become ragged from deep ulcerations, the strength of the acid may of course be greater, and I have often used it with equal parts of acid and water, but the introduction of the acid into the cervix of this strength causes so much pain as to necessitate the recumbent position and rest for some days thereafter. This acid I apply with the applicator, around the extremity of which had previously been laid a coating of cotton; this cotton is well wetted with the acid and quickly applied. Another remedy, and a favorite one, is the tr. iodine, with equal parts of cod liver oil or with glycerine. Sol. of subsulphate of iron is most valuable, but the permanent stains upon the instrument or upon whatsoever it comes in contact, makes it a somewhat disagreeable remedy to use. These remedies proving so satisfactory the nitrate of silver has almost fallen into disuse in our hands, although it is no doubt one of the most valuable remedies of this class.

I think I should hesitate a long time, and exhaust the materia medica in search of a remedy and exhaust my practice too, before resorting to the actual cautery. It appears like a barbarous remedy, if it be not such; and the civilization of our time would be much less shocked, and science receive greater homage and respect by limiting the use of this method of treatment to the brute creation, and forbearing to use the actual cautery as a remedial agent for diseases of the female sexual organs. I may entertain this much of disrespect for the use of the cautery, and at the same time acknowledge its usefulness in certain instances. Did I not make this acknowledgment what has been herein written, would indicate disrespect to the teachings of Ricord, and others, whose opinions are always entitled to consideration.

Mention might be made of other agents of this class, but this article has already exceeded the limits proposed for it in the outset, and must be brought to a close. Nothing could be gained by going through the whole catalogue of remedies; some one or all of which might be made available, although not possessed of equal merits.

As pertinent to the question of imposition I recall to mind now an extraordinary case of skilled diagnosis by a popular physician at the head of a "water cure establishment." He possessed the astonishing ability of looking through the speculum into the cavity of the uterus, through the uterus into the peritoneal cavity, and there beholding the work of destruction which had been going on for years, and explained to the satisfaction of his patient that certain bands within the abdominal cavity as a result of previous disease, had so much contracted as to draw down the whole abdominal viscera into a heap, which, of cousre, was the cause of her ailment, and that the treatment he should adopt would expand these bands and restore the parts to their former condition. The most extraordinary thing connected with this extraordinary diagnosis is, that the aforesaid physician made a most intelligent and cultivated lady believe in his absurd statement. On examination of this lady some time afterwards we found simply a retroverted womb.

The community of women, much more than members of the medical profession, are, or should be, interested in the subject matter of how they may enjoy immunity from medical imposition. In the absence of institutions managed by a corps of competent and skilled physicians, these women seek, as a dernier resort, a "cold water cure," and are at once put upon a course of treatment for uterine disease extending over a period of months. If perchance one escapes this treatment, it is only an exceptional case. If the physician in charge be competent and honest, all is well, but if he be incompetent, then upon the poor uterus rests the curse; then is the unfortunate uterus made to pay tribute to ignorance and the penalty for the overweening and misplaced confidence of its possessor.

It is time the profession had awakened from apparent lethargy; there is pressing necessity that the profession bestir itself, not indeed so much on account of itself, as on account and in behalf of those who are victims of misplaced confidence — of those who annually congregate at these popular places of resort for medical treatment. The profession should at once take this large class of invalids out of the hands of pretenders, by creating healthy public sentiment, and inciting the community to the importance of establishing a *place of refuge* adequate to the exactions of the opulent and the necessities of the poor.

